# Pathways through care of severely mentally ill individuals experiencing multiple public crisis events: a qualitative description

**DOI:** 10.1186/s12888-016-0787-8

**Published:** 2016-04-01

**Authors:** Mariëtte J. Hensen, Liselotte D. de Mooij, Jan Theunissen, Jack Dekker, Michael Willemsen, Jeroen Zoeteman, Jaap Peen, Matty A. S. de Wit

**Affiliations:** Public Health Service Amsterdam, Department of Epidemiology, Health Promotion, and Care Innovation, Amsterdam, The Netherlands; Arkin Mental Health Care, Research department, Amsterdam, The Netherlands; GGZ Ingeest Mental Health Care, Amsterdam, The Netherlands; Public Health Service Amsterdam, Department of Public Mental Health Care, Amsterdam, The Netherlands; Arkin Mental Health Care, Department of Emergency Psychiatry, Amsterdam, The Netherlands

**Keywords:** Crisis intervention, Severe mental illness, Pathways through care, Mental health care, Public mental health care

## Abstract

**Background:**

Patients experiencing severe mental illnesses (SMI) need continuing support and remain vulnerable in many domains. Crisis interventions and compulsory admissions are common, causing a huge burden on police, health workers, the community and patients. The aim of this retrospective case-file study is to determine profiles of SMI-patients and their pathways through care among those experiencing multiple public crisis events.

**Methods:**

Data from a larger study of 323 SMI-patients in Amsterdam were used. These data were linked to data of the public mental health care (PMHC) in order to identify persons that experienced crisis interventions (CI’s) between January 2004 and November 2012. The cut-off point for inclusion in the study population was set on three CI’s, resulting in a group of 47 SMI-patients. PMHC and mental health care (MHC) data were linked in order to identify profiles in patterns of care. Qualitative content analysis was used to gather and analyze chronological timelines.

**Results:**

Three profiles were identified: SMI-patients with CI’s during continuous MHC, SMI-patients with CI’s after discharge and SMI-patients with CI’s during unstable MHC. For each profile events prior to, during and after a CI were identified.

**Conclusions:**

PMHC and MHC can possibly identify cases with a high risk of CI’s and predict these events based on the results of this study. CI’s seem inevitable for a group of SMI-patients in care but they do not only require acute psychiatric care. The collaboration between MHC, PMHC and police could be further developed in a quick and effective triage in order to tackle the complexity of problems of the SMI-patients.

## Background

Severe mental illnesses (SMI) are associated with a complex mixture of clinical and social needs [1]. SMI-patients are often defined in relation to their long-lasting treatment situation, including criteria concerning the persistent nature of the disease (operationalized as >2 years of care) and an indicator of dysfunctioning (e.g. GAF score of less than 50) [2, 3]. SMI constitute only a minority of all mental illnesses; in many cases of mental illness dysfunctioning is mild and not chronic. Due to the chronic nature of SMI, continuing support from Mental Health Care (MHC) is crucial. The study on long-term care dependent patients (LZA-study) in Amsterdam found that the estimated amount of SMI-patients in care increased from 3000 in the year 2000 to 4576 patients in 2005 [4]. This increase was not explained as an increase in the total number of patients, but as in increase in the percentage of patients that was in treatment by the MHC; i.e. a better coverage by the MHC institutions among SMI-patients. In the past decades, the care for SMI has changed from long-term institutionalization to ambulatory care in the community and in sheltered housing. The current treatment is aimed at treating SMI-patients in an ambulatory setting instead of a clinical setting. Outreaching treatment teams, such as ACT (assertive community treatment) have become more and more common and since this increases the ambulatory treatment options, (compulsory) admissions are less often necessary and can often stay short.

Although treatment opportunities for SMI-patients have improved over the years, some patients still experience periods of social decline, and their behavior can cause nuisance to others. Of all long-term mental health patients in Amsterdam, 4–6 % experienced a crisis MHC contact outside office hours annually i.e. five crisis MHC contacts per 1000 inhabitants from 2000 to 2004 [5]. Although part of the SMI-patients function well with adequate treatment, the group remains vulnerable. For a small group of patients crisis interventions and compulsory admissions are common, the quality of life is below average, unmet needs remain present and suicide rates are up to nine times that of the general population [6, 7]. It seems that their social vulnerability, their complex needs of care, poor treatment adherence, comorbidities, and an unstructured existence, impede the way for providing the right care and even influence it negatively [8, 9].

The expected effects of dehospitalization in the ′80s and ′90s were: less restriction resulting in less aggression, more commitment from the social network, and a better (re-)integration into society. However, there were also fears, like the risk of overburdening the social network, increased use of medication and an increase in crisis MHC contacts [10]. Several publications report the incidence and risk factors for crisis MHC contacts within the mental health care system [5, 11–13]. Crisis MHC contacts are generally divided between those within office hours and those outside of office hours.

In Amsterdam, in the ′90s, similar to the situation in London [14], the fears seemed to have become reality. The public complained about overt psychiatric behavior and the associated nuisance. Dutch media reported an increase of problems in the streets that were clearly caused by people suffering from psychiatric disorders, such as paranoid or uninhibited sexual expressions, but also signs of deterioration [4, 10]. In addition psychiatric emergency units were overburdened. The psychiatric crisis chain in Amsterdam was reorganized in response, and the public health service got an active role in the coordination and screening of psychiatric patients [5, 15], [Raat, Municipality of Amsterdam, 2001].

The police gets involved in most crisis situations that develop outside of the scope of MHC and result in a public expression, mainly notified by third parties like other citizens, neighbors or housing corporations. In the past years, the Safetynet department of the public mental health service (PMHC) pre-screened all persons that experience a public crisis event and for whom the police suspects psychiatric problems. The number of these pre-screens has increased over the years, from 5 to 9 per 1000 adult inhabitants per year from 2003 to 2013 [Research and statistics department of the city of Amsterdam, Safetynet]. This might be due to the fact that a request for these consultations became more and more part of the regular process among the police. About 30–40 % of these contacts result in a referral to a crisis MHC contact within the unit for emergency psychiatry (SPOR), this percentage has remained fairly stable over the years. Those not referred to SPOR result in a variety of other services including social care or are followed by judicial trajectories.

These crises in the public domain have a large impact on the client, the public and the police. Little attention has been given to these public expressions and interventions in the literature. A study in Groningen about the role of the police in mental health crisis situations [16] showed that half of the individuals in crisis were disengaged from MHC in the year prior to the crisis. A body of literature is focused on factors that are related to (the re-occurrence of) psychiatric emergencies within MHC. Some studies shed light on socio-demographics and show an increased risk for migrants [17, 18] and those with a small social network [11]. Other studies focus on the influence of continuity of services and transitions, and show that a change of service provider, discharge from a clinic, are associated with an increased risk [8, 19–21]. However, most of these studies only included persons that were compulsory admitted after a crisis.

In this article we describe how public crises develop, what events precede them, and what characteristics of care surround them, in order to identify leads for prevention of crises and possibly better cooperation between the involved parties. The aim of our retrospective case-file study is to determine profiles of SMI-patients and their care among those experiencing multiple public crisis events in the period from 2004 to 2012. The collaboration between the MHC and PMHC for this group has not been described previously. Due to the chronicity of the illness, long-term treatment is needed and therefore patterns of care including the duration and intensity should be studied over a longer period of time [22]. A chronological overview of the characteristics of public crisis events and the offered help for these individuals provides insight into the frequency of crisis interventions in relation to characteristics of pre- and post-events and treatment. Altogether these findings make it possible to identify high risk groups with multiple crisis events and predict crisis situations and may contribute to the prevention of crisis situations.

### Description of the mental health care system in Amsterdam for psychiatric patients in crisis

Mental Health Care (MHC) and addiction care for SMI patients in Amsterdam are financed by the health insurance. In addition to regular services, MHC has several outreaching systems. It started with the rehab team that looked for vulnerable people anywhere in the city and if necessary, tried to persuade them to accept care. This later changed into teams working according to the assertive community treatment methodology [23]. Within office hours each institution operates their own crisis unit, outside office hours a central crisis unit is operational called SPOR [24]. The addiction care has a separate crisis unit, the clinical detoxification unit (CODA). At SPOR a (resident) psychiatrist decides whether or not a client needs to be admitted (compulsory or voluntary) [11, 15].

An acute compulsory admission in the Netherlands can be ordered in an acute crisis situation to avoid danger resulting from a psychiatric disorder. The procedure requires a psychiatrist to assess the patient. Based on the psychiatric condition and the danger to be averted, the mayor decides whether to assign the ACA. Within 3 days of admission to a mental hospital, a judge decides on the prolongation of the measure, by maximally 3 weeks. For a long term compulsory admission a decision of a judge is required before the start of the admission. These admissions have a duration of 6 months, after which a prolongation can be requested through court. Another possibility is discharge under court-ordered terms, such as the obligation to undergo treatment.

Clients could be referred to a crisis unit by medical doctors and by social psychiatric nurses (SPN’s) from the Safetynet department of PMHC.

The PMHC is financed by the city and intervenes when vulnerable people with mental and social problems are not able to provide for basic needs themselves and are not able or willing to organize the care they need to function in society in the opinion of professional care givers. Since 2006, integrated care is offered by cooperations between PMHC, shelters, MHC, addiction care, social services and the welfare agency. For each client an individual treatment plan is made, and a casemanager responsible for the coordination of care is assigned. The PMHC Safetynet department responds to reported crisis situations of vulnerable people. The SPN’s make an inventory of the situation and organize care, or coordinate existing care. Annually, about 2000 of these situations are reported. In addition, more than 5000 crisis signals came from the police who either responded to a signal from citizens, or responded directly to a situation they encountered in the public domain involving a vulnerable person. They could request an immediate consultation from a SPN 24 h a day, to decide which trajectory should be followed. In about 40 % of these crisis interventions, the client was sent to the SPOR. Some were sent to CODA. The rest was either sent home, with a referral to regular care or stayed to undergo the judicial consequences of their actions.

The situation concerning crisis signals from the police as described above was the situation up to December 2014. Since 2015 the police can directly refer patients to the SPOR and CODA. SPN’s from the Safetynet can be consulted in case of doubt. The situation in this article therefor describes the null-situation, to which future data can be compared [25].

## Methods

### Study group and data collection

This study targets SMI MHC patients that needed a crisis intervention of the PMHC between January 2004 and November 2012. To identify this group of SMI-patients, a selection of individuals was made from a larger study of SMI- patients in Amsterdam, the LZA-study.

The LZA research in 2005 studied 323 SMI-patients, with a history of MHC in the past 2 years. The objective of this study was to obtain information about quality of life, disease characteristics, general functioning, needs of care, social network and inclusion in society, and victimization of the 323 SMI- patients [10, 26]. Data were obtained through patient interviews, interviews with their doctors or nurses, and patient files. The data were linked to the digital client-registration system of the PMHC. Of the total sample of 323 people, 92 persons experienced one or more crisis interventions (CI’s) from Safetynet between January 2004 and November 2012. Patients with multiple CI’s were included in this study. Due to practical reasons and to maintain the feasibility of this qualitative research, the cut-off point of the group experiencing multiple CI’s was set on three. This resulted in a group of 47 SMI-patients.

#### Data sources

PMHC: All reported (crisis) interventions from Safetynet are described in a journal in the registration system. The journals contain extended information about the intervention and were studied to obtain the following themes: date of CI, location, notifier, description of the crisis, action and results of Safetynet interventions. All information presented in the results section of the study came directly from these journals, including judgment of the situation and reasons for action. No additional interpretation was made by the researchers. Journals were manually coded in SPSS 19 by the first author and discussed with the second and last author. Extended information was extracted from journals of reported signals (e.g. decompensation etc.) and from reported regular notifications of care and nuisance.

The regular registration system of the MHC-institutions (PSYGIS) including the patient files, were consulted to obtain chronological information about MHC; type of treatment, department, and type of treatment contacts.

LZA study: In order to describe characteristics of the 47 people in the study group, data were collected from the initial interviews from the LZA study (described above).

Municipal population register: source of information for changes in address and time lived in Amsterdam, as an addition to the LZA data.

#### Data analysis

According to the cross case method, analysis was based on document analysis of the group with multiple crisis reports established by Safetynet (*N* = 47). The process was iterative and exploratory, not all variables were determined in advance. PMHC and MHC data were linked into personal chronological timelines in order to identify longitudinal pathways of care. All information from the Safetynet journals that described the events before, during and after a CI was incorporated. These combined descriptions were coded, based on themes from the literature and emerging themes, such as: continuity of care, follow up contacts, avoiding care, medication adherence, times between CI, signals preceding CI. These coded timelines were studied by two researchers independently, who each defined emerging profiles, based on similarities in the nature of a CI, the re-occurrence of CI and the continuity of MHC. After the two researchers reached consensus, the profiles were checked on face-validity by the second and third author. After identifying profiles, the personal chronological timelines were studied in order to discover cohesion between events.

## Results

Of the 323 SMI-patients included in the original study population, 47 (15 %) experienced multiple crisis interventions by PMHC, and formed the study population (Table 1). The majority consisted of men with a Dutch origin. One fifth of the total group had a Surinam origin. The median age was almost 42 years, with a range of 23 to 62. At baseline, more than half of the population received ambulatory care and more than one third resided in a psychiatric clinic. Most people were diagnosed with schizophrenic disorder. From more than half of the group it was known that they were drugs and/or alcohol dependent, at some point during the study period.

**Table 1:** Characteristics of 47 severely mentally ill individuals who experienced 3 or more crisis interventions

	Profile 1		Profile 2		Profile 3		Total	
	*n* = 10	%	*n* = 15	%	*n* = 22	%	*N* = 47	%
Gender								
Male	5	50 %	9	60 %	18	82 %	32	68 %
Female	5	50 %	6	40 %	4	18 %	15	32 %
Age (median)	41,4		44,5		41,3		41,9	
Cultural Origin								
Dutch	4	40 %	12	80 %	13	59 %	29	62 %
Other	6	60 %	3	20 %	9	41 %	18	38 %
Mental health treatment at baseline								
Clinic	2	20 %	9	60 %	5	23 %	16	34 %
Sheltered housing	0	0 %	2	13 %	4	18 %	6	13 %
Ambulant	8	80 %	4	27 %	13	59 %	25	53 %
Homeless (at least once during study period)	1	10 %	3	21 %	12	57 %	16	36 %
Changes of address (median)	1,7		3,4		5,6		3,6	
DSM Primary diagnosis								
Psychotic disorder	2	20 %	0	0 %	3	14 %	5	11 %
Schizophrenic disorder	7	70 %	14	93 %	12	54 %	33	70 %
Bipolar disorder	1	10 %	1	7 %	0	0 %	2	4 %
Dissociative disorder	0	0 %	0	0 %	1	5 %	1	2 %
Alcohol and/or drugs dependency	0	0 %	0	0 %	6	27 %	6	13 %
DSM II Personality disorders	2	20 %	2	13 %	5	23 %	9	19 %
Substance abuse^a^	3	30 %	10	67 %	16	73 %	29	62 %
GAF (median score)	48		40		55		40	

^a^based on all data: DSM diagnose, MHC type of care and Safetynet journals

### Profiles

The process of studying the available data from PMHC and MHC resulted in the identification of three profiles. Table 2 describes the care characteristics that turned out to best distinguish these profiles.

**Table 2:** Description of the different profiles based on PMHC journals of SMI-patients that experienced multiple CI’s in the period from 2004–2012

Features profiles	Nature of primary MHC	Continuity of MHC	Event for CI	Nature of CI	Reoccurrence of CI
Profile 1 ‘CI during continuous MHC’	Ambulatory	Stable	Signs of decompensation/medication non adherence, public nuisance towards neighbors	Psychological, problems in the social domain	Extended periods without CI
Profile 2. ‘CI after discharge clinic’	Clinical	Change in frequency	discharge from institution	Severe, danger for others/themselves	Multiple CI’s until compulsory admission
Profile 3 ‘CI during unstable MHC’	Ambulatory addiction	Unstable, few/no contacts, care avoiders	Signs of self neglect	Substance abuse, deterioration	Long periods without CI

The first profile, “CI during continuous MHC”, consists of ten clients who were frequently and continuously in contact with the MHC and had a relatively stable life for extended periods of time. The dominant type of MHC for this profile throughout the study period was ambulatory. Substance abuse was low in this profile, and men and women were equally represented. The majority had a non-Dutch origin (Table 1). The second profile, “CI after discharge clinic”, consists of 15 clients with very complex problems experiencing severe crises. The dominant type of care throughout the study period for this profile was clinical care. Some persons were admitted throughout the whole study period (*n* = 6) and experienced CI’s during a period of clinical care. The continuity of care differed per period. A crisis mainly occurred (within one month) after discharge or during free time from the clinic. The patients in this profile were slightly older, two thirds had problems with substance abuse and they had the lowest GAF score of the three profiles (average GAF-score of 40). The third profile, “CI during unstable MHC”, consisted of 22 clients who lacked continuous contact with the MHC. Almost half of this profile (*n* = 10) did not have any MHC contacts before a CI occurred in the study period. This profile consisted mainly of men, and the majority had substance abuse problems. Dominant type of care through the study period was ambulatory care concerning substance abuse. Instability was also observed in housing, with a high average number of address changes. Results will be discussed by theme per profile: before the CI, the CI itself and after the CI.

### Profile 1: crisis during continuous MHC

#### Before the crisis intervention

Contacts: An increase in crisis MHC contacts in the month before a CI was observed several times. In some cases the CI’s were preceded by a change in regular ambulatory contact frequency (increase or decrease) with MHC.

Signals to PMHC: In the weeks before a CI, sometimes the MHC reported official signals to the PMHC with concerns about decompensation, psychotic symptoms and/or medication non-adherence. These signals were meant to inform Safetynet because of the large possibility that they would be called in to intervene and to request referral of these clients to the SPOR if they came into contact.

Events: From the journals of the CI’s it appeared that this profile often caused public nuisance towards their neighbors (e.g. aggressive or agitated expressions towards neighbors or noise) prior to a CI.

#### The crisis intervention itself

Description of the incident: Most CI’s of this profile started at the client’s home, mainly reported by the police and the “care and nuisance” department of the city of Amsterdam. However, CI’s in the public domain also occurred frequently. Some CI’s were reported in institutions or at police stations where clients occasionally show up by themselves. Reasons for a CI were mainly nuisance at home or in the streets but also problems in the social domain like self-neglect, neglect of the household, domestic violence or eviction. At clients’ homes the nuisance involved threatening neighbors or household members, throwing furniture outside and screaming. In some cases clients came to the police office themselves (confused) to complain about their neighbors or to report theft or missing items. Nuisance in the streets included psychotic behavior (e.g. disordered; anxious; restless; agitated), screaming and/or yelling, threatening other people. In addition, showing signs of self-neglect were also reported. A reason for a CI could also just be a reaction to an official signal and request of the MHC to refer a client to SPOR.

Action from Safetynet: Safetynet often responded by making house visits in in order to judge the situation. In most of these cases, the client was referred (back) to their treating physician, therapist or nurse at the MHC. Other cases resulted in a referral to SPOR by Safetynet. Sometimes the CI only involved organizing care in cooperation with other parties, like social services, and putting more pressure on the client to solve the problem (like cleaning the house). Most CI’s that started in the streets, resulted in a referral to SPOR.

#### After the crisis intervention

Result of the CI: Referrals to SPOR often resulted in a compulsory admission with varying durations. In other cases, problems in the social domain escalated before the situation improved (e.g. concerns of rent arrears and neglected households into threatening eviction), resulting from the fact that clients did not follow the advice voluntarily and could not be forced. Sometimes also, an organization delayed action after signaling a problem (e.g. threatening eviction reported by a housing corporation to the MHC). The CI was followed by a continuation of the regular ambulatory care pattern in all cases. Discharge from the clinic was followed by MHC contacts within days. The time before re-occurrence of a CI varied between months and years. New CI’s mainly occurred due to another period of decompensation.

### Profile 2: Crisis after discharge clinic

#### Before the crisis intervention

Contacts: In several cases an increase in crisis MHC contacts was observed in the week prior to a CI or a decrease in the months before.

Signals made to PMHC: In the period shortly before a CI, several official signals were reported by the MHC to the PMHC. The signals for this profile involved concerns about decompensation, substance abuse, aggressive behavior and withdrawal of conditions of court authorizations. In almost all of these cases, MHC requested PMHC to refer the client to SPOR as soon as they were in the picture.

Events: A CI for this profile mainly occurred within 1 week after discharge or during free time from a psychiatric clinic. In many cases people were discharged due to the fact that the compulsory admission or court authorization expired. Sometimes clients got suspended from the clinic because they were unmanageable (e.g. threatening staff and household members or vandalism). Some cases included frequent police contacts due to nuisance (towards neighbors) in the short period between discharge and the CI (before the police decided to call in Safetynet).

#### The crisis intervention itself

Description of the incident: Most CI’s started in the street and involved clients with confused, psychotic, threatening and (sexually) disinhibited behavior (e.g. masturbation, walking naked and defecating in public). At home, the CI’s often involved clients that caused nuisance immediately after discharge from the clinic, like; screaming, threatening neighbors, causing fire and throwing furniture into the streets. Some clients contacted the police to report theft or because they were afraid in their own homes (after discharge). Several clients tried to enforce re-admission through these public expressions or tried to claim certain care by threatening with suicide. Some clients refused to identify themselves or gave up a false name.

Action from Safetynet: In most cases clients were referred to SPOR by Safetynet. In cases where clients were unmanageable or disrupted an institution, Safetynet provided an alternative through a referral to SPOR. On occasions, the CI concerned a client that ran away from the clinic, who was not reported missing, Safetynet identified a client or clarified the housing situation, and clients were sent back to the clinic by the police or sometimes on their own.

#### After the crisis intervention

Result of the CI: Most clients in this profile were referred to SPOR and (re-)admitted to a clinic. However, in many cases clients were also sent away by SPOR because of a lack of severe psychiatric symptoms at that moment. It sometimes took multiple CI’s and acute compulsory admissions before a court authorized admission was (re-)achieved. A new CI re-occurred within 1 week in many of these cases (some within hours). In one case voluntary admission was rejected by the clinic due to capacity problems despite SPOR arguing for their admission.

### Profile 3. Crisis during unstable MHC

#### Before the crisis intervention

Contacts: In many cases, there were less than six contacts in the 6 months prior to a CI. Almost half of the profile did not have any contacts at all before the first CI in the study period.

Signals made to PMHC: The signals for this profile, prior to a CI, mainly involved concerns about increased self-neglect and substance (ab)use, reported by the MHC or social workers. It could take months before Safetynet met a client experiencing a CI after such a signal.

Events: Some clients were suspended from night shelters prior to a CI. CI’s also occurred within hours or days after clients were sent away by SPOR. In cases, related to alcohol abuse, it appeared that the drinking increased due to relational or family problems (e.g. not allowed to see children, thrown out of the house by partner). In one case admission to the crisis detoxification unit (CODA) was rejected by MHC, days prior to the CI.

#### The crisis intervention itself

Description of the incident: The reason for a CI was mainly public nuisance followed by self-neglect, and deterioration of psychiatric symptoms. Public nuisance in this profile involved: aggressive behavior; begging, substance abuse and sleeping in the streets, resulting in many police contacts. In many cases the police found clients in the streets roaming, deteriorated, desperate or drunk. One client got evicted from his house which immediately caused an increase of CI’s. In many other cases clients reported themselves at the police station. Clients that appeared at the police office or called the emergency number were afraid, paranoid, asked for shelter or reported theft or violence. The clients who only experienced CI’s related to their alcohol abuse (*n* = 3), mainly reached out when they were completely desperate, sometimes they were arrested very drunk in combination with suicidal expressions. During their CI’s, they often tried to enforce admission to CODA. Sometimes Safetynet got involved, after failed attempts by MHC to contact a client.

Action from Safetynet: The CI’s that started in the street lead to referrals to SPOR, referrals to the MHC (outreach workers), or admission to CODA. Most clients that showed up at the police station were referred to SPOR or Safetynet arranged night shelter. Clients that caused nuisance at home were mainly referred to SPOR. In some cases the client was sent away without any concrete actions. Some clients refused further care at that moment.

#### After the crisis intervention

Result of the CI: Some clients denied the presence of psychiatric problems, refused MHC contacts, refused medication or refused to give their (correct) identity. In several cases, CI’s did not result in an admission but only in actions by the criminal justice system. This was the case when the SPOR concluded that the crisis did not result from acute psychiatric symptoms, but was related to behavioral problems or lack of sleep or exhaustion. In some of these cases another CI occurred within weeks due to psychotic problems. The clients that did get admitted in a psychiatric clinic were often there for a longer period, months or years. CI’s related to alcohol abuse often resulted in admission to CODA. In many cases, the admissions ended prematurely because the client refused appointments or ran away and refused aftercare. It often took months to several years before a new CI occurred with clients showing up with a cry for help. This turn of events repeated itself again and again over the years. For some clients the continuity of MHC transferred from none to frequent contact (weekly) at some point in the 8 years of follow-up. In many cases (*n* = 13) this lead to an improved and/or stable situation in the end. Even after years of deterioration, CI’s, lacking MHC contacts; improvement could still be achieved through admission to a clinic and an increase of ACT contacts and care.

## Discussion

Of the total study group of 323 SMI-patients, 92 had one or more crisis interventions by the PMHC in a period of 9 years, and 47 had three of more PMHC crisis interventions in this period (15 %). Three distinct profiles could be distinguished within this group.

The first profile existed of SMI-patients with ongoing MHC contacts, the second profile experienced CI’s mainly after discharge from the psychiatric clinic and the third profile lacked continuous MHC contacts.

### Profile 1: Crisis during continuous MHC

Results show that even stable contacts with MHC cannot always prevent the occurrence of decompensation (mainly due to medication nonadherence) and social problems. Although some CI’s were preceded by an increased frequency of contacts and signals from family, friends and neighbors, the actual occurrence of CI’s was not prevented in this profile. Often because voluntary interventions did not result in improvement, and because compulsory interventions are only possible when the threat of danger is clear. In these cases, the current system does not seem to provide for more preventive action and since some periods of decompensation seems to be inevitable, compulsory admission seems to be an effective way to restore a stable situation. A more preventive approach might be feasible for crisis situations in the social domain. In some cases, the home situation had deteriorated severely in spite of regular MHC contacts. From this study, we cannot conclude whether more preventive action was feasible, nor whether the lack of motivation of the client hampered all possible interventions, but the fact that signals of an upcoming crises are picked up is a first prerequisite for prevention. Especially since these CI’s often occur at home, and the Dutch care system is switching more towards integrated care in the neighborhoods. In different neighborhoods, collaborations between different partners are being created, depending on the local problems, and care infrastructure. These collaborations support vulnerable client in society, by focusing not only on their mental health, but also on all other domains of life, including housing, finances, and participation in society. Possibly these collaborations might prevent escalation by early signaling in the home situation or staying in contact with family and friends and the availability of multiple leads for intervention.

### Profile 2: Crisis after discharge clinic

This profile experienced severe crisis events and interference of PMHC was frequently necessary. CI’s were often preceded by increased crisis MHC contacts, official signals from the MHC and discharge from a psychiatric clinic. It seems that the subjects in this profile are not able to cope outside the clinic. Discharge is often due to withdrawn court authorizations. CI’s seem to be a tool for police, MHC and PMHC to obtain a court order but legal regulations often hampered this. The transition to compulsory admission is based on the degree of danger resulting from psychiatric symptoms. Often this was not an option and a proper alternative was lacking, resulting in new CI’s within days and creating a huge burden for the police and other parties involved, leaving clients very vulnerable. For some severely mentally ill persons, compulsory admission and long term clinical care seem the only solution within the treatment options at the time of the study. They are not able to cope outside a psychiatric clinic, with or without ambulatory care. A high number of previous admissions are a predictor for readmission within 6 months [27]. In the light of the current trend to decrease the number of beds in the clinics and switch to more ambulatory care, the burden on and of patients in this profile might increase. New alternatives should be sought to provide sufficient support for patients in this profile outside the clinical setting, but with intensive outreaching guidance, strong motivational expertise, and possibilities for preventive coercion.

### Profile 3: Crisis during unstable MHC

CI’s were often related to the marginal living situation and circumstances of these clients, due to drugs and/or alcohol dependence and/or homelessness. The need for care often arose at the (crisis) moment. Due to these circumstances, the care during a CI was not always focused on psychiatric problems, these problems were not directly visible. No appropriate care seems to exist for the clients in this profile who, in general, feel no need for care or even actively avoid care. Their sudden upcoming demand for care, that diminishes within days, renders mental health care without sustainable options, and the motivation of MHC and addiction care to offer short term solutions whenever the clients demands these, decrease each time a care trajectory or clinical episode is prematurely ended by the client. It is clearly difficult to achieve and maintain frequent contact within this profile of whom many are alcohol and/or drug dependent. Based on the available data, it is impossible to tell how much effort was put in to maintaining contacts. This study showed however that CI’s provide an opportunity to resume or start MHC care and contacts. For more than half of the individuals within this profile, increased ACT (after admission) resulted in improvement of the situation. ACT also reduces the likelihood of disengagement from services [28]. Therefore it seems important to keep putting effort in reaching out to this profile of possible care avoiders, even though they constitute only a small proportion of all SMI-clients.

### Strengths

By combining data from three large institutions (two MHC and PMHC) that provide care for SMI-patients in Amsterdam, we were able to provide longitudinal insight in patterns of care. Reviewing every patient journal in detail made it possible to extract much information about crisis interventions. This made it possible to determine profiles of a group of SMI-patients experiencing multiple CI’s. With these results, the PMHC and MHC can possibly identify cases and predict crisis situations.

### Limitations

The data from the Safetynet system come from an administrative database. All details of psychiatric emergencies were made in free text by a large panel of professionals from the department and may not be as accurate and complete as those made with diagnostic research instruments and structured interviews. Second, although our data included a period of almost 9 years, this period did not coincide with the start of a care trajectory. This may have limited the insight in pathways of care. Also, the long follow-up period of the study inevitably also included changes in the organization of care, and the introduction of new interventions, such as Assertive Community Treatment (ACT). In addition, we may not have covered all MHC contacts. For example, the data did not include contacts with MHC outside the region, contact with private mental health practices, or contacts with prison mental health services. Finally, the study population focused on the subgroup of patients with multiple CI’s. A quantitative evaluation of the whole group is in progress and will provide additional insight into factors in which this problematic groups differs from a group in which less public interventions were necessary. Since it is an observational study, we provide insight in the current process in which MHC, PMHC and the police work closely together. However, we could not compare this to different situations in which one of the parties was absent or other options in interventions were available. It might therefore be very insightful to conduct similar analyses in other cities or countries with different systems as a comparison.

### Leads for prevention

The study had two aims; to identify high risk groups and predict crisis situation(1) and contribute to the prevention of these situations (2). High risk groups seem most readily definable by developments in a patient’s situation over time instead of personal characteristics. These developments include changes in the availability of important persons in the social network, like the hospitalization or death of a parent; a change in housing situation, which includes discharge from a clinic; and personal deterioration or deterioration of the living environment. In order to use these signals for prevention, they need to be picked up and considered to be important by the involved professionals, who then need to have options to act. Especially this last step seems difficult, since it often involves people not motivated for voluntary options and the situation is not yet severe enough for involuntary options. Motivational techniques, involving the social network when available, therefore seem to be a key element in prevention of crises. In addition, patients might feel a need for support in other domains than mental health, such as housing or shelter or hygiene. When this support is provided by professionals with an integral approach, this might contribute to prevention as well. Outreaching care with unplanned house visits forms an important element in timely signaling and also helps in maintaining the relationship between patient and care professional in times of low motivation of the patient.

A few more specific leads for preventing crises became apparent. These are not new, but are confirmed by these results: intensive and immediate follow-up after discharge from a psychiatric or addiction clinic [29], the value of signals of concerned family members, control of medication adherence. It also needs to be said that some crises cannot be predicted and prevented, underlining that a public response remains necessary.

## Conclusions

The general conclusion is that the interference of parties in the public domain seems necessary for a small group of SMI-patients that are already known and in treatment by the MHC. The results showed that the police and PMHC play an important role in signaling crisis situations and referring individuals to the MHC, but also in referring clients to other types of care and support. Where public mental health care has an important role in picking up signals and referring to different organizations, the police has an important role in signaling problems in the public and domestic domain and in recognizing that these problems might be related to underlying mental or social problems. The base of this collaboration between MHC, PMHC and police could be further developed in order to tackle the complexity of the SMI-patients. Since many cases do not require acute psychiatric care, it seems that quick and effective triage is essential to decide which care system should be involved. Several problems emerge from this study, differing by subgroup. Medication non-adherence is a problem in all profiles, especially in the first profile. Continuity of care, including continuation of care after discharge from the clinic (aftercare) but also prolongation of clinical care are important issues in the second profile. Motivation, outreaching contact, and prevention of deterioration emerge as the most important issues from the third profile. Creative interventions and cooperations seem necessary to provide effective care for all three of these profiles of complex chronic patients, especially in the light of the current new wave of de-institutionalization in the Netherlands.

## Declarations

### Ethical approval and consent to participate

The LZA study was approved by the Medical Ethical Committee of GGZ InGeest Mental Health Care and the Medical Ethical Committee of the Free University in Amsterdam and also covers the current study which falls within the research aims of the LZA-study. The study population gave written informed consent for using their data for scientific research and link their data to databases used in this study. Based on this permission, access to the data of the registries of the MHC-sytems, the PMHC-system and the municipal register was granted.

No individual data was used in this manuscript (only aggregate data are presented). Because of the small sample in combination with detailed information, the underlying data cannot be made available due to privacy reasons.

### Consent for publication

Not applicable.

## Abbreviations

SMI: severe mental illness
MHC: Mental Health Care
PMHC: Public Mental Health care
LZA-study: study on longterm care dependent patients
CI: crisis intervention (by the public mental health care department)
Psygis: client register of mental health care
GAF: global assessment of functioning
SPOR: unit for emergence psychiatry
CODA: clinical detoxification unit
SPN: social psychiatric nurses
